# Beyond “all-or-nothing” climbing fibers: graded representation of teaching signals in Purkinje cells

**DOI:** 10.3389/fncir.2013.00115

**Published:** 2013-07-02

**Authors:** Farzaneh Najafi, Javier F. Medina

**Affiliations:** ^1^Department of Biology, University of PennsylvaniaPhiladelphia, PA, USA; ^2^Department of Psychology, University of PennsylvaniaPhiladelphia, PA, USA

**Keywords:** complex spike, cerebellum, mossy fiber, motor learning, calcium, LTD, dendrite, motor learning

## Abstract

Arguments about the function of the climbing fiber (CF) input to the cerebellar cortex have fueled a rabid debate that started over 40 years ago, and continues to polarize the field to this day. The origin of the controversy can be traced back to 1969, the year David Marr published part of his dissertation work in a paper entitled “A theory of cerebellar cortex.” In Marr’s theory, CFs play a key role during the process of motor learning, providing an instructive signal that serves as a “teacher” for the post-synaptic Purkinje cells. Although this influential idea has found its way into the mainstream, a number of objections have been raised. For example, several investigators have pointed out that the seemingly “all-or-nothing” activation of the CF input provides little information and is too ambiguous to serve as an effective instructive signal. Here, we take a fresh look at these arguments in light of new evidence about the peculiar physiology of CFs. Based on recent findings we propose that at the level of an individual Purkinje cell, a graded instructive signal can be effectively encoded via pre- or post-synaptic modulation of its one and only CF input.

Marr’s idea that cerebellar climbing fibers (CFs) play the role of “teachers” during motor learning was a stroke of genius. Like the rest of the hypotheses first introduced in his revolutionary “A theory of cerebellar cortex” (Marr, 1969), the idea that CFs provide instructive signals was built from the ground up, based on first principles and a deep understanding of the computational problems that need to be solved in motor control. In addition, Marr relied extensively on detailed knowledge about the wiring circuit and the physiology of the cerebellar cortex, which had been compiled just a few years before in a remarkable book by Eccles et al. (1967). We may never know with certainty what led to the aha moment that sparked the idea that CFs could act as “teachers”; but one can only imagine that in developing his pioneering theory, Marr must have been particularly intrigued by the unique properties of the CF input and the peculiar response it generates in the post-synaptic Purkinje cell.

## “ALL-OR-NOTHING” INSTRUCTIVE SIGNALS

**FIGURE 1:**
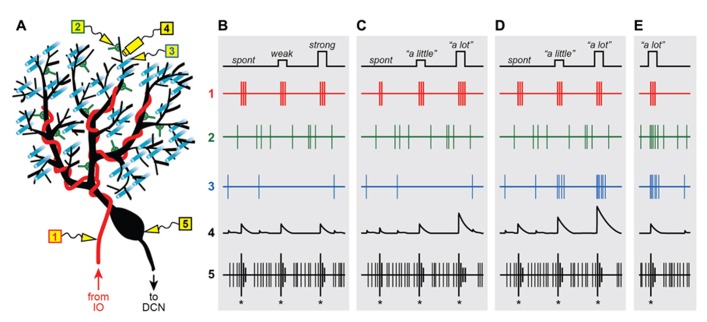
** Graded instructive signals in a Purkinje cell.** **(A)** A schematic diagram of a Purkinje cell and its different synaptic inputs. Electrodes are placed in different locations to measure the extracellular spiking activity of the climbing fiber (1; red), a molecular layer interneuron (2; green), a parallel fiber (3; cyan) and the Purkinje cell axon (5; black). In addition, intracellular calcium signals are imaged in one of the Purkinje cell’s distal dendrites (4; black), near electrodes 2 and 3. **(B–E) **Spikes and calcium signals measured in the five locations shown in **(A)**, under four different scenarios: when all climbing fiber signals are “all-or-nothing” whether firing is spontaneous (spont) or when the strength of stimulation in the inferior olive is weak or strong **(B)**, when instructive signals to lift the foot “a little” or “a lot” influence the number of spikes in the climbing fiber burst **(C)**, or when the instructive signals activate the climbing fiber simultaneously with parallel fiber inputs **(D)** and input from the molecular layer interneurons **(E)**.

Climbing fibers are the axons sent by neurons in the inferior olive to the contralateral cerebellum (**Figure 1A**; red; Eccles et al., 1966; Desclin, 1974; Schmolesky et al., 2002; Ohtsuki et al., 2009). One of the most striking features of this olivo-cerebellar projection is that in the adult cerebellar cortex, each Purkinje cell is innervated by a single CF (Eccles et al., 1966; Schmolesky et al., 2002; Ohtsuki et al., 2009). This is one of the most powerful excitatory synapses in the brain (Eccles et al., 1966), comprising more than 1000 contacts distributed all along the proximal portion of the Purkinje cell dendritic tree (Palay and Chan-Palay, 1974; Strata and Rossi, 1998). As a result, activation of a single olivary neuron results in a large electrical event in the soma of the post-synaptic Purkinje cell, termed the “complex spike” (CS; Thach, 1967) because it consists of a fast initial spike followed by several slower spikelets of smaller amplitude separated from each other by 2–3 ms (**Figure 1B5**; asterisk; Eccles et al., 1966). The CS can be easily distinguished from the so called “simple spikes” (Thach, 1967), normal action potentials fired constantly by the Purkinje cells at high rates (**Figure 1B5**; thin lines). The cause of the spikelets in the CS was disputed for years (Armstrong and Rawson, 1979; Campbell et al., 1983b), but recent work has demonstrated that they are a result of the interaction between local resurgent sodium currents in the Purkinje cell soma (Raman and Bean, 1997, 1999a, 1999b; Schmolesky et al., 2002), and the characteristic activation of the pre-synaptic CFs, which tend to fire in brief high-frequency bursts of 1–6 spikes (**Figure 1B1**; Crill, 1970; Armstrong, 1974; Mathy et al., 2009).

From the very beginning, the somatic CS was described as being “all-or-nothing” (Eccles et al., 1966), a label that has stuck to this day. This characterization of the CS is based on the finding that direct microstimulation of the inferior olive causes a seemingly binary response in the post-synaptic Purkinje cell (Eccles et al., 1966): “nothing” if the strength of stimulation is below a certain threshold, or a unitary (“all”) CS for all strengths above threshold (**Figure 1B5**; same CS for weak or strong inferior olive stimulation). In other words, the CS evoked in an individual Purkinje cell is unaffected if additional CFs are activated by increasing the strength of stimulation in the inferior olive.

These groundbreaking experiments hold an important place in history, partly because in showing that the post-synaptic CF response does not depend on the number of stimulated cells in the inferior olive, they helped demonstrate that each Purkinje cell must receive input from one-and-only-one CF (Eccles et al., 1966). Importantly, the “all-or-nothing” quality of the post-synaptic CS also implies that the response of the sole pre-synaptic CF input is not graded with the strength of olivary stimulation, and must itself be “all-or-nothing” as well (**Figure 1B1**; same 3-spike burst for weak or strong IO stimulation). Later studies confirmed this prediction by recording directly from individual neurons in the inferior olive, and showing that their spiking response varies little with the strength of stimulation (Crill, 1970). This finding has far-reaching implications and is at the center of a heated debate about the functional role of the CF input.

## SPONTANEOUS CLIMBING FIBERS AND THE SIGNAL-TO-NOISE PROBLEM

To Marr, the idiosyncratic properties of the olivo-cerebellar system could only mean one thing: each individual “all-or-nothing” CF input represents an “elemental” instruction that provides information about what the correct movement should be in a given context (Marr, 1969). It is important to remember that in the original theory, these instructive signals could be encoded in either motor or sensory coordinates (Marr, 1969). For example, if an obstacle is placed in front of the right foot causing the subject to trip while walking on a treadmill, the appropriate elemental instruction could be represented using motor commands coming from cerebral cortex (e.g., “lift right foot”), or sensory-related inputs coming from peripheral activation of cutaneous receptors (e.g., “the right foot hit an obstacle”). In either case, the idea was that the CF input would be providing an instructive signal to the Purkinje cell, triggering mechanisms of plasticity that would be used to correct subsequent movements (i.e., lift the right foot higher on the next step cycle and avoid the obstacle).

Almost 45 years after Marr’s original proposal, his hypothesis remains controversial and the cerebellar field is still divided with regards to how CF signals are used to exert control over our movements (De Schutter and Maex, 1996; Simpson et al., 1996; Llinás, 2011). It appears that at least in some motor learning tasks, CFs are activated in a manner that is compatible with their presumed role as “teachers” (Gilbert and Thach, 1977; Raymond et al., 1996; Simpson et al., 1996; Kitazawa et al., 1998; Raymond and Lisberger, 1998; Ito, 2006, 2013; Medina and Lisberger, 2008; Rasmussen et al., 2008; Soetedjo et al., 2008). Further support comes from *in vitro *studies showing that CF inputs can trigger a variety of synaptic plasticity mechanisms in Purkinje cells (for reviews, see Hansel et al., 2001; Gao et al., 2012). However, a number of questions have been raised about the potential instructive role of CFs during motor learning, particularly with regards to the problems inherent in representing information with “all-or-nothing” signals from spontaneously active neurons (Llinás and Welsh, 1993; Llinás et al., 1997).

One argument against the idea that CFs act as “teachers” is that the “all-or-nothing” CF input is ambiguous from the point of view of an individual Purkinje cell, and suffers from the so-called “signal-to-noise” problem (Llinás et al., 1997). The trouble is that CFs are spontaneously active about once per second (Armstrong, 1974; Simpson et al., 1996), and at least in the prevailing view (**Figure 1B**), the post-synaptic Purkinje cell would have no way of distinguishing between these frequent spontaneous activations (“noise”), and the few which occur during motor learning and presumably encode elemental instructions (“signal”). Even if Purkinje cells were somehow able to discriminate between instructive and spontaneous CF inputs, the “all-or-nothing” character of the CF signal would put a hard limit on how much information can be encoded. At best, a CF could fire (“all”) to signal “lift right foot” or remain silent (“nothing”) to signal “do not lift right foot,” but it would not be able to provide useful parametric information about how far to lift it. These theoretical considerations call into question the ability of individual CFs to provide efficient instructive signals for motor learning. But are CFs really such “bad teachers?”

## POOLING TOGETHER CF SIGNALS: THERE IS STRENGTH IN NUMBERS

Previous theoretical studies have suggested that even though a single “all-or-nothing” CF signal is ambiguous, an individual Purkinje cell could still solve the “signal-to-noise” problem by collecting information from its CF input across many trials (Sejnowski, 1977; Fujita, 1982; Kawato and Gomi, 1992; Gilbert, 1993; Mauk and Donegan, 1997; Mauk et al., 1997; Kenyon et al., 1998; Spoelstra et al., 2000; Dean et al., 2010). In these computational models, CF activity works as an equilibrium point signal: the CF fires (“all”) to trigger plasticity when an error is made and the movement needs to be adjusted, but is silent (“nothing”) if the movement is performed correctly. Because a single spontaneous CF input cannot be distinguished from a single error-related CF input, it is assumed that both types of CF signals are equally capable of inducing plasticity. However, only those CF signals that are repeatedly triggered with high probability in a specific learning context would lead to an enduring change in the Purkinje cell. This solves one problem, but leaves unanswered one important question: how can “all-or-nothing” CFs provide parametric information about the size of the error? After all, an effective instructive signal should indicate whether the movement requires just a small adjustment or a major overhaul.

An “all-or-nothing” CF signal cannot carry much information by itself, but instructive signals with details about error size could be encoded, at least in theory, by pooling together the activity of many olivary neurons. For example, the instructive signal “lift right foot” could be represented by activating any one of ten CFs, while at the same time graded information about how far to lift it could be encoded by modulating how many of the ten are simultaneously activated. The olivo-cerebellar system seems perfectly suited for this type of synchronous population coding: neighboring neurons in the inferior olive are electrically coupled by dendrodendritic gap junctions (Llinás et al., 1974; Sotelo et al., 1974; De Zeeuw et al., 1996, 1997; Marshall et al., 2007), and as a result, small groups of CFs converging on the same narrow parasagittal strip of cerebellar cortex have a tendency to fire synchronously (Bell and Kawasaki, 1972; Llinás and Sasaki, 1989; Sugihara et al., 1993; Simpson et al., 1996; Lang et al., 1999; Kitazawa and Wolpert, 2005). Furthermore, the level of co-activation in the CF population appears to encode sensorimotor-related information (Lou and Bloedel, 1992; Welsh et al., 1995; Wylie et al., 1995; Lang, 2002; Ozden et al., 2009; Schultz et al., 2009; Wise et al., 2010).

As pointed out by others (Ozden et al., 2009; Schultz et al., 2009; Bengtsson et al., 2011; Otis et al., 2012), the level of CF co-activation could potentially be read out and used as an instructive signal in downstream neurons of the deep cerebellar nuclei which receive convergent input from many Purkinje cells (Palkovits et al., 1977; Person and Raman, 2011). However, our concern here is with the representation of instructive signals at the level of an individual Purkinje cell, which receives input from a single CF (Eccles et al., 1966; Schmolesky et al., 2002; Ohtsuki et al., 2009), and therefore does not have easy access to information encoded in the population. Note that in theory, a Purkinje cell could receive information about activation of neighboring CFs through spillover mechanisms (Szapiro and Barbour, 2007; Mathews et al., 2012), but this possibility will not be considered further in this paper. Instead, we will discuss alternative ways to enhance the information capacity of individual olivary neurons, using mechanisms that challenge the conventional view that all CF signals are created equal.

## MODULATION OF THE PRE-SYNAPTIC CLIMBING FIBER BURST

New discoveries about the spike-generating mechanisms of olivary neurons are challenging conventional wisdom about the way CFs encode information. As noted earlier, CFs fire in brief high-frequency bursts, comprising 1–6 spikes separated from each other by 2–3 ms (Crill, 1970; Armstrong, 1974; Mathy et al., 2009). The burst is generated in the olivary axon itself, as a result of an intrinsic positive feedback loop (Mathy et al., 2009): the first spike is initiated in the axon, but it also backpropagates into the dendrites where it opens high-voltage-activated calcium channels that cause a prolonged depolarization lasting up to 10 ms. When this depolarization reaches the axon, it triggers the rest of the spikes in the burst.

At first glance, this seemingly automatic and self-driven burst mechanism appears to fit well with the “all-or-nothing” character of the CF response to brief olivary stimulation (Crill, 1970), which was mentioned earlier and is characterized by a single burst of spikes that varies little whether the initial depolarization is just above threshold or much stronger (**Figure 1B1**). However, it is known that the processes underlying spike generation and dendritic depolarization are both influenced by a variety of factors, including the resting potential of the inferior olivary neuron (Llinás and Yarom, 1981; Ruigrok and Voogd, 1995). This opens up the possibility that information may be transmitted by modulating the number of spikes in the CF burst.

Indeed, the era of the “all-or-nothing” CF may be coming to an end. Recent studies have shown that the burst size, i.e., the number of spikes in the CF burst, is tightly regulated and provides extra information not available in the conventional binary signal (Maruta et al., 2007; Mathy et al., 2009; Bazzigaluppi et al., 2012; De Gruijl et al., 2012). For example, burst size is correlated with a number of critical parameters which together define the state of olivary neurons. These cells have a characteristic subthreshold membrane potential oscillation which is synchronized across neighboring olivary neurons via gap junctions (Lampl and Yarom, 1993, 1997; Devor and Yarom, 2002; Leznik and Llinás, 2005). It has been shown that the number of spikes in the CF burst varies systematically according to the phase of the oscillation *in vitro* (Mathy et al., 2009), the amplitude of the oscillation *in vivo* (Bazzigaluppi et al., 2012), and the extent of electrotonic coupling and synchrony in a computer model of the olivary network (De Gruijl et al., 2012). In addition, burst size can be used to distinguish between spontaneous and sensory-related CF signals evoked by sinusoidal whole-field visual stimulation (Maruta et al., 2007). This last study also found that the number of spikes in the CF burst varied systematically depending on the direction of the visual stimulus.

The findings of the studies mentioned in the preceding paragraph must be interpreted with some caution. As was also the case in previous experiments (Eccles et al., 1966; Armstrong and Rawson, 1979), the number of spikes per CF burst was quite variable from one burst to the next and always fell within the same limited range (1–6 spikes), regardless of condition or behavioral state. Therefore, the changes in burst size for any given situation were small (<1 spike per burst) and could only be detected in the average as a slight probability bias toward generating more bursts with many (>4) or few (1) spikes. It remains to be seen whether such a fickle modulation of the CF-burst signal could play a functional role during motor learning, perhaps by regulating the induction of plasticity in the post-synaptic Purkinje cell (Mathy et al., 2009). Nonetheless, these groundbreaking experiments have demonstrated that the number of spikes in the CF burst is not entirely random and can thus provide parametric information not available in a binary code.

**Figure 1C** illustrates a straightforward way to encode a graded instructive signal by systematically modulating the number of spikes in the CF burst, e.g., 2 spikes for “no instruction” due to spontaneous activation, 3 for “lift right foot a little,” and 4 for “lift right foot a lot.” Clearly, this example is an oversimplification. In reality, codes based on burst size would be inherently noisy because as mentioned above, the number of spikes in the CF burst is subject to stochastic variations within a limited range. However, the information capacity of an individual CF would still be enhanced under conditions in which burst size is probabilistic and only slightly biased one way or another depending on the parametric details of the instruction. A similar proposal for encoding parametric information in the CF system was formulated on theoretical grounds almost 40 years ago (Gilbert, 1974).

One advantage of the code in **Figure 1C1** is that it can be unambiguously read-out because a difference of just one spike in the CF burst has a substantial impact on the response evoked in the post-synaptic Purkinje cell. In the dendrites, burst size regulates the duration of the depolarizing plateau potential (Campbell et al., 1983a), the number of calcium spikes (Mathy et al., 2009), and the ability of the CF input to induce plasticity (Mathy et al., 2009; **Figure 1C4**). With regards to Purkinje cell output, burst size has a strong influence on both the number of CS-related spikes that are sent down the axon (Mathy et al., 2009), and the duration of the characteristic pause in simple spike activity that follows the CS (Mathy et al., 2009; **Figure 1C5**).

## MODULATION OF THE POST-SYNAPTIC CLIMBING FIBER RESPONSE

It is often overlooked that the same groundbreaking paper that coined the term “all-or-nothing” to describe the Purkinje cell CS also made it very clear that the excitatory post-synaptic potential (EPSP) evoked after activation of the CF input could itself be graded (Eccles et al., 1966): the size of the EPSP was shown to depend critically on the membrane potential. This observation has important implications for the coding of instructive signals in Purkinje cells, particularly as it pertains to the regulation of CF-evoked calcium influx in the dendrites.

Activation of the CF input causes a massive depolarization of the proximal dendrites of the Purkinje cell (Eccles et al., 1966), triggering regenerative calcium spikes that propagate and cause calcium influx throughout the dendritic tree (Ross and Werman, 1987), including the terminal spiny branchlets (Konnerth et al., 1992; Miyakawa et al., 1992), where the excitatory parallel fiber (PF) synapses are located (**Figure 1A**; cyan). Dendritic calcium is the trigger for a wide variety of short-term (Batchelor and Garthwaite, 1997; Glitsch et al., 2000; Brenowitz and Regehr, 2003; Maejima et al., 2005; Rancz and Hausser, 2006) and long-term (Sakurai, 1990; Konnerth et al., 1992; Kano et al., 1996; Hansel and Linden, 2000; Miyata et al., 2000; Wang et al., 2000; Coesmans et al., 2004; Tanaka et al., 2007) mechanisms of plasticity in Purkinje cell synapses, and for this reason it is considered the neural implementation of behaviorally driven instructive signals at the most fundamental molecular level (for reviews, see Hansel et al., 2001; Gao et al., 2012).

What is important about the CF-triggered dendritic calcium signal from a neural coding perspective is that just like the evoked EPSP, its amplitude can be modulated *in vitro* (Miyakawa et al., 1992; Midtgaard et al., 1993; Callaway et al., 1995; Wang et al., 2000) and *in vivo* (Kitamura and Häusser, 2011) by a variety of factors that influence the membrane potential of the Purkinje cell. For example, activation of inhibitory synapses from molecular layer interneurons (**Figure 1A**; green) causes a conductance shunt that reduces the amplitude of the CF-triggered calcium signal (Callaway et al., 1995). Conversely, dendritic calcium influx is significantly enhanced if the CF input is preceded by stimulation of the excitatory PF pathway (Wang et al., 2000), which by itself causes a small graded calcium response via activation of voltage-gated calcium channels as well as metabotropic receptor-dependent release from intracellular stores (Eilers et al., 1995; Takechi et al., 1998).

**Figure 1D** illustrates a straightforward way to encode a graded instructive signal by systematically modulating the amplitude of the CF-triggered calcium response in the Purkinje cell dendrites. The three signals corresponding to “no instruction” due to spontaneous activation of the CF input, “lift right foot a little” and “lift right foot a lot” are associated with progressively increasing levels of PF excitation (**Figure 1D3**), and as a result, they are encoded in the dendrite as progressively larger calcium responses (**Figure 1D4**). Note that in this example there is a parallel systematic modulation of the characteristic post-CF pause in Purkinje cell activity (**Figure 1D5**), which is consistent with the recently described effect of extra dendritic calcium spikes on somatic spiking (Davie et al., 2008). On the other hand, the CS itself provides no parametric information about the instruction because it is the same regardless of the context in which the CF was activated (**Figure 1D5**). This is consistent with previous work demonstrating that the burst pattern of the CS is largely unaffected by dendritic events because the CF input causes a functional division between dendritic and axosomatic compartments (Davie et al., 2008).

We have made one key assumption in **Figure 1D**: the instructive signal that activates the CF input also activates some of the PF synapses on the same Purkinje cell. In other words, our proposal requires a high degree of spatial convergence in the cerebellar cortex: PF’s and CFs inputs representing the same type of information must come together at the level of individual Purkinje cells.

The field is currently divided with regards to this “convergence” hypothesis (Apps and Garwicz, 2005). Previous studies have provided irrefutable evidence that the CF receptive field of an individual Purkinje cell matches that of the mossy fibers located in the granular layer directly underneath (Garwicz et al., 1998; Brown and Bower, 2001; Voogd et al., 2003; Odeh et al., 2005; Pijpers et al., 2006; Apps and Hawkes, 2009). What is less clear is whether this vertically aligned spatial organization would result in the Purkinje cell receiving the CF signal together with excitatory input from mossy fiber-driven PF’s (Cohen and Yarom, 1998; Brown and Bower, 2001; **Figure 1D**), or with inhibitory input from mossy fiber-driven molecular layer interneurons (Ekerot and Jorntell, 2001, 2003; **Figure 1E**). Based on classic work (Eccles et al., 1967, 1972; Eccles, 1973), as well as more recent studies using *in vivo* imaging of peripherally evoked inhibitory responses in the cerebellar cortex (Gao et al., 2006) or patchy photostimulation of granule cells *in vitro* (Dizon and Khodakhah, 2011), we think both scenarios are possible. We suspect that the levels of local excitatory and inhibitory input may differ between groups of Purkinje cells, depending on their precise location relative to the activated PF’s. This raises the intriguing possibility that the mossy fiber pathway may be used to set the membrane potential of the Purkinje cell, and in this way adjust the efficacy of CF-related instructive signals.

## EPILOG: CF-DRIVEN PLASTICITY IN PURKINJE CELLS

Our paper highlights how graded modulation of individual CF inputs may be used for encoding parametric information about instructive signals. But to really understand the role of CFs in motor learning, we must first answer one fundamental question: if CFs are the “teachers,” who might the students be and what do they learn? In “A theory of cerebellar cortex,” Marr predicted that CFs would teach by modifying the strength of excitatory PF synapses (**Figure 1A**; cyan). Immediately after the publication of his revolutionary theory, Marr himself worked with Eccles on this topic, but “failed to discover any significant modification even after some hundreds of parallel fiber-climbing fiber inputs” (Eccles, 1973). This initial failure did not stop others from trying to induce plasticity by stimulating CFs with more physiological patterns. More than a decade later, Masao Ito would become the first person to demonstrate CF-dependent long-term depression (LTD) of PF synapses (Ito and Kano, 1982). Since then, research about cerebellar plasticity has exploded (Gao et al., 2012). We now know that CFs can trigger a variety of long-term modifications in PF synapses (**Figure 1A**; cyan; Ito and Kano, 1982), in molecular layer interneuron synapses (**Figure 1A**; green; Kano et al., 1992; Duguid and Smart, 2004; Mittmann and Häusser, 2007), and even in the CF synapse itself (**Figure 1A**; red; Hansel and Linden, 2000; Bosman et al., 2008; Ohtsuki and Hirano, 2008). The functional significance of these plasticity mechanisms remains largely unknown. We can only imagine that in contrast to the conventional “all-or-nothing” instructive CF input, the type of graded CF signals we have described here could provide an extra degree of flexibility for choosing carefully who the students are and what to teach them.

## Conflict of Interest Statement

The authors declare that the research was conducted in the absence of any commercial or financial relationships that could be construed as a potential conflict of interest.
